# Soil Particle Size Thresholds in Soil Spectroscopy and Its Effect on the Multivariate Models for the Analysis of Soil Properties

**DOI:** 10.3390/s23229171

**Published:** 2023-11-14

**Authors:** Issam Barra, Tarik El Moatassem, Fassil Kebede

**Affiliations:** Center of Excellence in Soil and Fertilizer Research in Africa (CESFRA), College for Sustainable Agriculture and Environmental Sciences (CSAES), Mohammed VI Polytechnic University (UM6P), Ben Guerir 43150, Morocco; tarik.elmoatassem@um6p.ma (T.E.M.);

**Keywords:** particle size, FTIR, soil properties, partial least squares regression

## Abstract

This study focused on one of the few but critical sample preparations required in soil spectroscopy (i.e., grinding), as well as the effect of soil particle size on the FTIR spectral database and the partial least squares regression models for the prediction of eight soil properties (viz., TC, TN, OC, sand, silt, clay, Olsen P, and CEC). Fifty soil samples from three Moroccan region were used. The soil samples underwent three preparations (drying, grinding, sieving) to obtain, at the end of the sample preparation step, three ranges of particle size, samples with sizes < 500 µm, samples with sizes < 250 µm, and a third range with particles < 125 µm. The multivariate models (PLSR) were set up based on the FTIR spectra recorded on the different obtained samples. The correlation coefficient (R^2^) and the root mean squared error of cross validation (RMSECV) were chosen as figures of merit to assess the quality of the prediction models. The results showed a general trend in improving the R2 as the finer particles were used (from <500 µm to 125 µm), which was clearly observed for TC, TN, P_2_O_5_, and CEC, whereas the cross-validation errors (RMSECV) showed an opposite trend. This confirmed that fine soil grinding improved the accuracy of predictive models for soil properties diagnosis in soil spectroscopy.

## 1. Introduction

Soil, defined as a thin layer that covers the Earth’s surface, is among the most important resources for human life with regard to food production [1]. Its importance emanates from its complexity, as it is considered as the most complicated biomaterial on Earth. Soil characteristics contrast spatially and temporally, making soil quality monitoring very challenging [2,3].

In 1951, Nelson et al. [4] showed that the rational use of agricultural soil analysis can contribute to better soil management. Modern diagnosis of agricultural soil began with Bray in 1948 [5], who developed analytical procedures to quantify soil nutrient reserves. Eight years later, Fitts and Nelson [6] suggested the use of soil testing results in fertilizer and liming recommendations.

Soil quality monitoring is a key task allowing the understanding of the nature (i.e., particle size distribution, acidity, and nutrient availability) and state of health of soil, which impact productivity and, thus, crop yield [7]. Nevertheless, the standard soil testing methods, which require numerous dangerous chemicals, are labor intensive, expensive, and slow. On the other hand, the recent approaches to simplify soil testing, especially with emerging international crises such as global climate change due to environmental pollution, prompts the use of green technologies such as infrared spectroscopy for both in-field and laboratory-based soil analyses, thus avoiding chemical reagents and chemicals. Spectroscopic techniques are considered as fast, low-cost, and reliable methods that can provide a consistent solution for rapid soil analysis.

In recent years, infrared spectroscopy in its two main ranges (NIR and MIR) has been increasingly used not only as a qualitative tool (i.e., molecular bounds identification), but also as a quantitative tool in many fields, namely, food, pharmaceutics, petroleum, and for soil diagnosis [8,9]. The coupling of spectral techniques with multivariate modeling algorithms (i.e., chemometrics and machine learning modeling) was found to provide a fast, cost-effective, environmentally friendly, and non-destructive solution, until becoming a trendy field of research and development for assessing various soil physical, chemical, and biological properties [10].

Among the advantages of this technology is the fact that it requires few steps to be performed before the analysis of samples, i.e., drying the soil sample at 39 °C for about 24 h and fine grinding [11]. However, the problem that can appear during preparation is the effort required to grind the soil particles very finely (<80–200 µm). Although it is costly and tedious, in order to synchronize the effective diameter of the soil sample (i.e., powdered sample) with the diameter of the infrared beam, which is generally between 1 and 2 mm, the particle should be as fine as possible in order to have as many units that contribute to the signal leading to the spectrum, and larger particles can cause specular reflections and yield spectra that do not appropriately represent the sample [11,12].

Numerous studies agree on the importance of finely grinding soil samples for mid-infrared spectroscopy analysis (MIR) as a solution for sample homogenization, inhibition of specular reflections, the elimination of spectral artifacts, and for increasing the amount of materials in contact with the light beams [11,12,13,14]. Conversely, some papers advise that too much grinding may have adverse effects on the resulting spectra, namely, the destruction of chemical bonds of soil organic or mineral constituents, for two main reasons, i.e., the mechanical effect and frictional heating [14,15].

Regarding the study of the effect of grinding the soil samples on soil spectroscopy models, Janik et al. (2016) [16] achieved a significant improvement for clay and sand models and impairments for silt. Guillou et al. (2015) [11] used 227 samples from Australia to test the effect of fine grinding on the quality of PLS models. The results showed that by using fine ground samples (<1 mm), the model accuracy was improved for organic C, sand, and clay, but no enhancement was noted for silt. Deiss et al. (2019) [17] worked on 400 soils from the U.S. and tested the influence of grinding on the quality of support vector machine regression models for five soil properties (i.e., clay, sand, permanganate-oxidizable C, pH, and TOC). Their finding was that the best models were obtained using fine ground simples < 0.5 mm with collecting multiple spectra (repetitions). 

In all previous studies, it was confirmed that fine soil grinding increased the accuracy of predictive models. The comparisons were made between spectra collected on ground soil samples and on raw samples, the gap that exists in the bibliography is that none of this research compared models resulting from different grinding ranges to decide on the best particle size choice to obtain the best machine learning model.

To address this issue, in the present paper, the effect of soil particle size was studied by comparing the resulting PLSR models from three ranges of particle size (<500 µm, <250 µm, and <125 µm) to conclude on the best practice to improve predictions for eight soil properties (TC, TN, OC, sand, silt, clay, Olsen P, and CEC).

## 2. Materials and Methods

### 2.1. Soil Sample Preparation and FTIR Spectra Acquisitions

#### 2.1.1. Soil Sample Preparation

**Figure 1:**
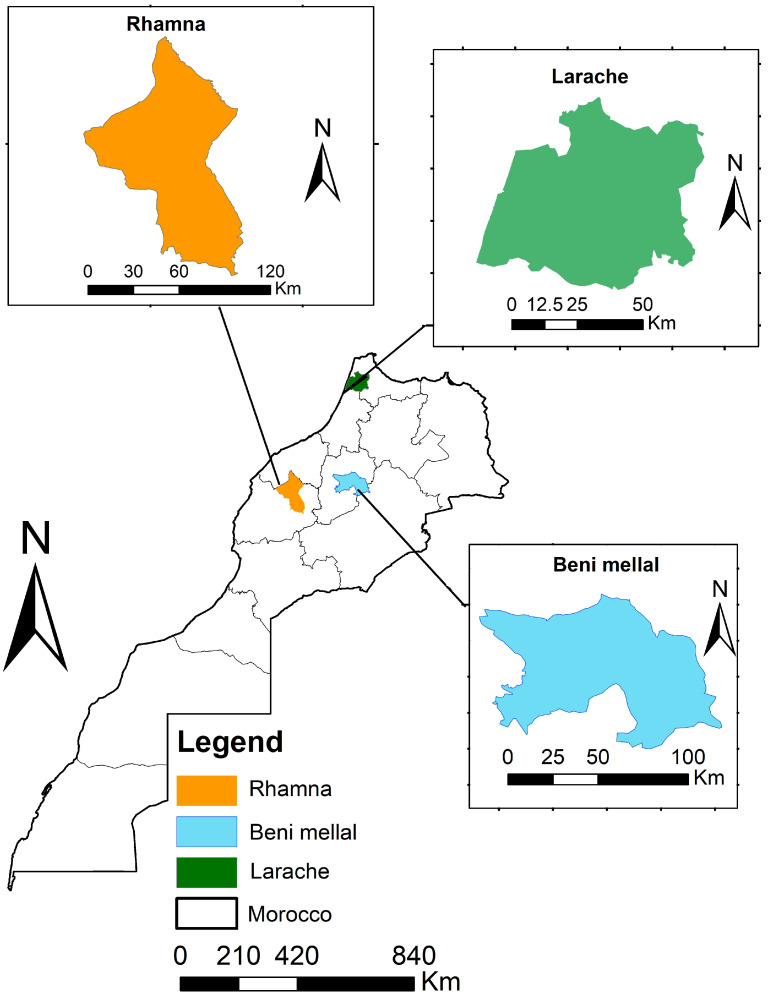
Location map of the study sites, viz., Rhamna, Beni Mellal, and Larache regions of Morocco.

**Figure 2:**
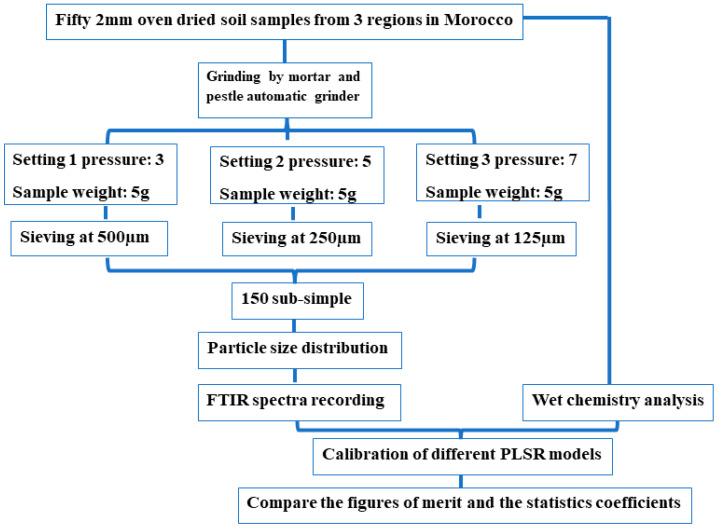
Workflow for building the database used in the study of the effect of particle size on soil spectroscopy.

The study was focused on soils of Morocco, which were collected from three agricultural regions, namely Rhamna, Larache, and Beni Mellal (see Figure 1). These three regions are among the largest agricultural regions with significant production, characterized by fertile and calcareous soils (Rhamna). A total of 50 soil samples were collected from the three regions taking into account soil textural variations. These soils were ground using a pestle and mortar to pass through a 2 mm sieve after drying in an oven at 39 °C for 48 h. Subsequently, the samples were ground further until it turned to powder using an automated pestle and mortar grinder RM200 from Retsch by applying three settings of pressure of the mortar and the pestle (i.e., 3, 5, and 7), as outlined in the workflow depicted in Figure 2. Then, the soil samples were sieved to pass through 500, 250, and 125 µm, resulting in 150 subsamples. 

#### 2.1.2. FTIR Spectra Acquisition

The soil powders were then loaded into a 96-micro-well spot plate adaptable for the Tensor autosampler. Two sub-samples from each sample were loaded into two wells on the plate. The surface of the sample in each well was flattened using a spatula to facilitate spectral scanning. The spectra recording was conducted between 4000 and 600 cm^−1^ with 4 cm^−1^ as resolution, and the scan number was set at 60 scans per acquisition [18], using a Bruker Tensor II bench-top spectrometer Massachusetts, USA at the Soil Spectroscopy Laboratory of the Centre of Excellence for Soil and Fertilizer Research in Africa, at the Mohammed VI Polytechnic University, Morocco.

### 2.2. Determination of Particle Size Distribution

After the sieving operation of the 150 subsamples, the particle size distribution of the soil samples was achieved using a Mastersizer 2000 from Malvern Instruments Ltd., Malvern, United Kingdom, in the range 0.02 to 2000 µm, using water as dispersant, to ensure that the size of soil particles respect the fixed ranges (<500, <250, and <125 µm), and to confirm the success of the grinding and sieving operation.

### 2.3. Soil Property Measurements

Soil physical–chemical properties such as soil total carbon (TC) via combustion method (ISO 10694), total nitrogen via combustion method (ISO 13878), organic carbon (OC) (ISO 10694), sand, silt, and clay (Bouyoucos method ISO 13317), available phosphorus using Olsen method (ISO 11263), and the hexamine-cobalt method for cation exchange capacity (NF ISO 23470) were analyzed in the Soil Testing Laboratory of the Agricultural Innovation and Technology Transfer Center (AITTC-UM6P).

### 2.4. Chemometrics Analysis

Chemometrics is defined as the part chemistry that integrates mathematical modeling and computer tools to highlight the valuable information from analytical data [19]. It is generally used to reduce data dimensionality and investigate the relationships between samples and variables [20,21].

Chemometric tools can be classified into two main types, viz., unsupervised methods (i.e., Principal Components Analysis), applied as exploratory methods, and supervised methods, used for predictive purposes [22,23].

Partial least squares (PLS) regression belongs to the supervised method category, and it is very widely used with spectroscopic data as the standard chemometric tool applied to perform calibrations and predictions [24]. PLS models the relationship between two matrices, X (spectroscopic data) and Y (variable to be predicted), by finding linear combinations of X and Y matrices that are called latent variables (LVs) [25].

In the present study, the effect of the sample’s particle size was assessed by setting-up predictive models using the entire FTIR spectra measured on the soil samples (X matrix) after all the preparation steps as described in Figure 2. To remove the external variation effects, the 1st derivative preprocessing was applied to all the calibrated models [26]. It is the simplest form of Savitzky–Golay derivatives applied to eliminate useless signals and reduce the scattering effect before the spectral data proceed to the calibration step. The validation of the built models was performed via the “leave one out” cross-validation method [27], which made it possible to calculate the figure of merit (R^2^ and RMSECV) required to assess the predictive quality of the PLS models.

### 2.5. Software for Data Processing and Statistical Criteria for Assessing the Quality of the PLSR Models

The performance evaluation of the PLS models was performed by testing two main figures of merit, namely, the cross-validation error or root mean squared error of cross-validation (RMSECV), calculated as:

(1) $$\mathrm{RMSECV}=\sqrt{\frac{\sum_{i=0}^{N}{\left({\hat{y}}_{i}-y_{i}\right)}^{2}}{N}}$$

where N is the number of samples in the calibration set, y_i_ and ŷ_i_ are, respectively, the observed and predicted values for sample I and the correlation coefficient R^2^ [28,29].

The calibration of the different PLSR models was performed on OPUS Quant II 8.1 software from Bruker Optiks GmbH, Billerica, MA, USA.

## 3. Results and Discussion

### 3.1. FTIR Spectra

**Figure 3:**
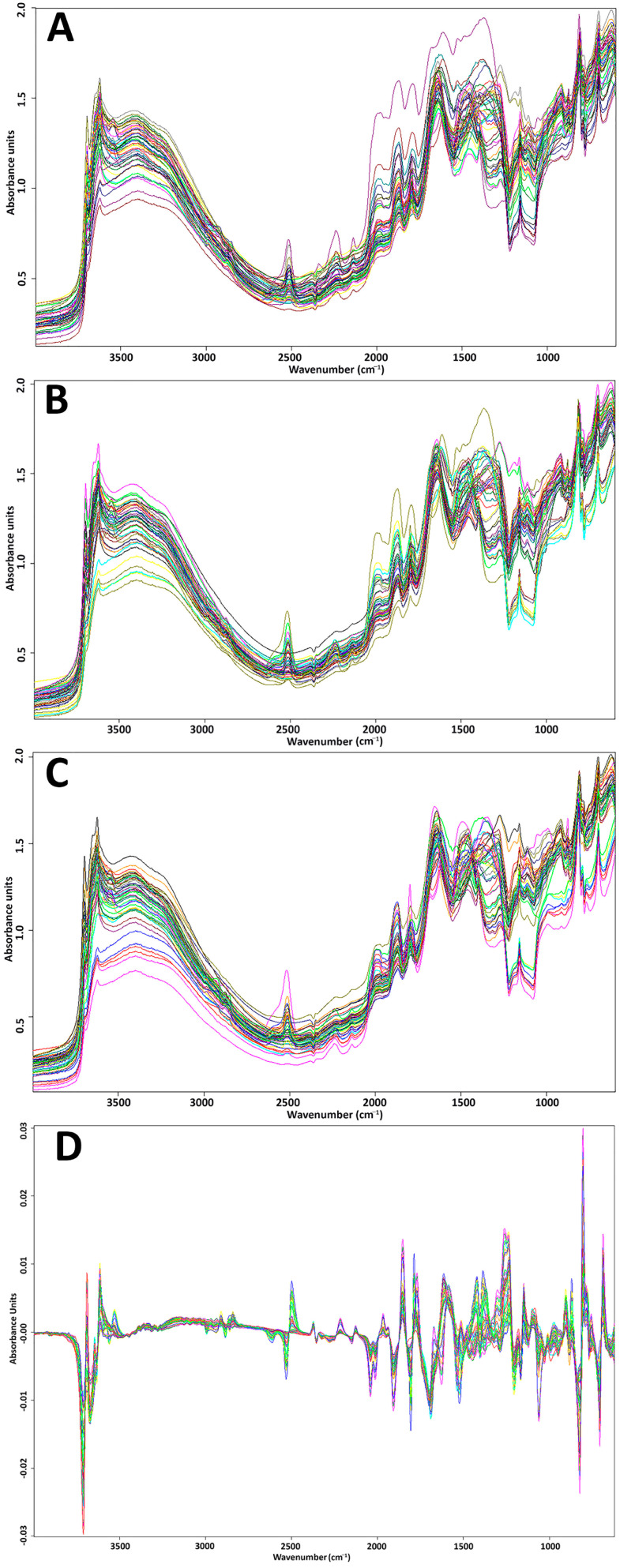
FTIR spectra of the soil samples ground and sieved at 500 µm (**A**), 250 µm (**B**), and 125 µm (**C**) in the mid-infrared range (600 to 4000 cm^−1^); (**D**) represents the total spectra after the preprocessing (first derivative). The different colors represent the different samples.

Figure 3 shows the different spectra obtained after grinding and sieving operations, where spectra A, B, and C denote the spectra of particle sizes 500, 250, and 125 µm, respectively. On these spectra, the fundamental vibrations between 4000 and 2500 cm^−1^ are caused by O–H, C–H, and N–H stretching; more precisely, the peaks around (3800–3600 cm^−1^) are attributed to O–H stretching in clay minerals [30], and the bonds near to 3550 cm^−1^ are associated with the Al-OH vibrations from kaolinite [31]. The region from 2500 to 2000 cm^−1^ is linked to the triple-bond stretching vibrations such as the nitrile group (C≡N), which can be observed between 2200 and 2300 cm^−1^. The double-bond vibrations can be found in the region between 2000 and 1500 cm^−1^, such as the C=C and C=O stretching observed in the range 1500–2500 cm^−1^ [32]. While the fingerprint is highlighted in the range between 1500 and 400 cm^−1^ [32], the interpretation of the different bounds in this region is difficult since it characterizes the fingerprint of the mineral compounds [30]. In addition, through visual inspection, no difference can be highlighted between the spectra belonging to the distinct groups of particle size.

On the other hand, after the application of the first derivative preprocessing (Figure 3D), the spectral data became more homogeneous, and the spectral abundance of the major bands is easier to highlight. This provides reassurance that the external effects and the noise have been eliminated, and only the main part relating to the chemistry of the samples is remaining.

### 3.2. Particle Size Distribution

**Figure 4:**
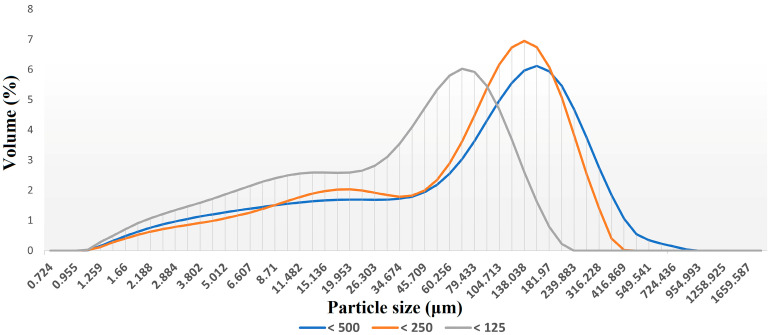
The average particle size curves of the samples belonging to the different particle size groups <500 µm, <250 µm, and <125 µm.

The determination of the particle size distribution of the different groups of samples prepared (ground and sieved) was performed in order to ensure that the particles size of these samples respect the fixed ranges (<500, <250, and <125 µm) before proceeding to the FTIR spectra recording. The results showed that the grinding and the sieving operations led to samples that respected the particle size limits; the abundance of the desired particle sizes is centered in the middle of the intervals (90% of the particles of the three groups <125, <250, and <500 µm have, respectively, a particle size in the range of 5–125 µm, 40–250 µm, and 50–400 µm, and no cases of contamination have been detected. Figure 4 represents the average particle size curves of the samples belonging to the different particle size groups.

### 3.3. Effect of the Particle Size Distribution on the Predictive Capacity of Soil Spectroscopy Models

**Figure 5:**
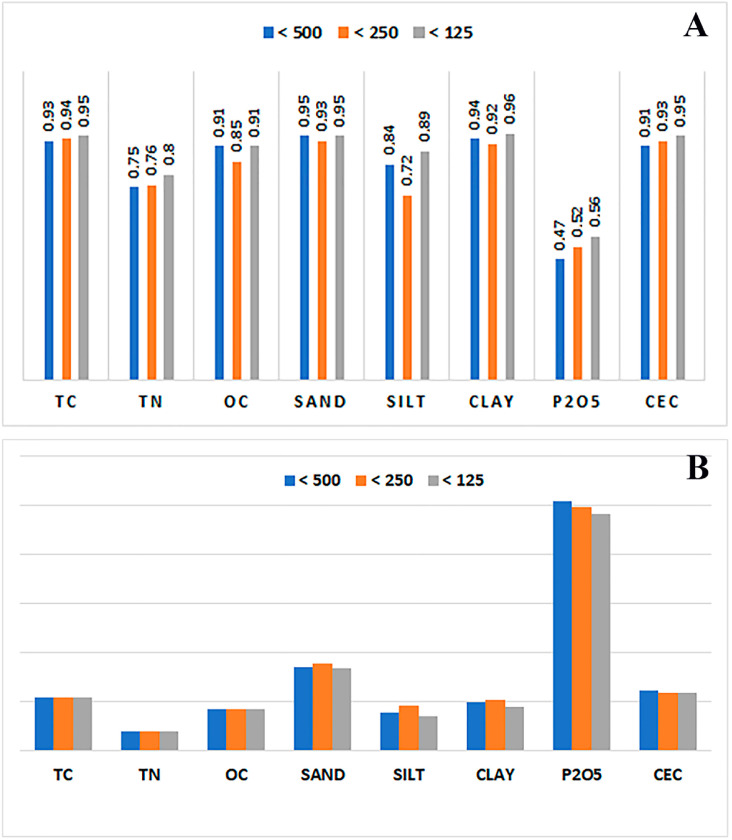
(**A**) Variation in the correlation coefficients according to the size of sample particles prepared before the acquisition of the FTIR spectra of the fifty soil samples. (**B**) Variation in the root mean squared error of cross validation (RMSECV) for eight selected soil properties according to the particle size range of the forty prepared soil samples.

To better highlight the effect of the soil sample’s particle size on the FTIR spectra and the predictive models, partial least squares regression was used to set up calibrations for each of the eight soil properties against the particle size range (i.e., <500, <250, and <125 µm) using a set of fifty soil samples. By inspecting the figures of merit of the different models set up for the eight soil properties, based on the samples resulting from the various preparation operations, a general increasing trend in the correlation coefficient R^2^ was observed from the models with samples <500 µm to samples <125 µm, since it was noticed that the models built on the finer particles were characterized with the highest R^2^ (Figure 5A); except for OC and the texture (sand, silt, and clay), it can be seen that the effect can be considered insignificant. In contrast, the opposite was observed for the cross-validation error RMSECV, which decreased when decreasing the size of the particles, as the >250 µm models were distinguished by producing the lowest errors (Figure 5B).

**Figure 6:**
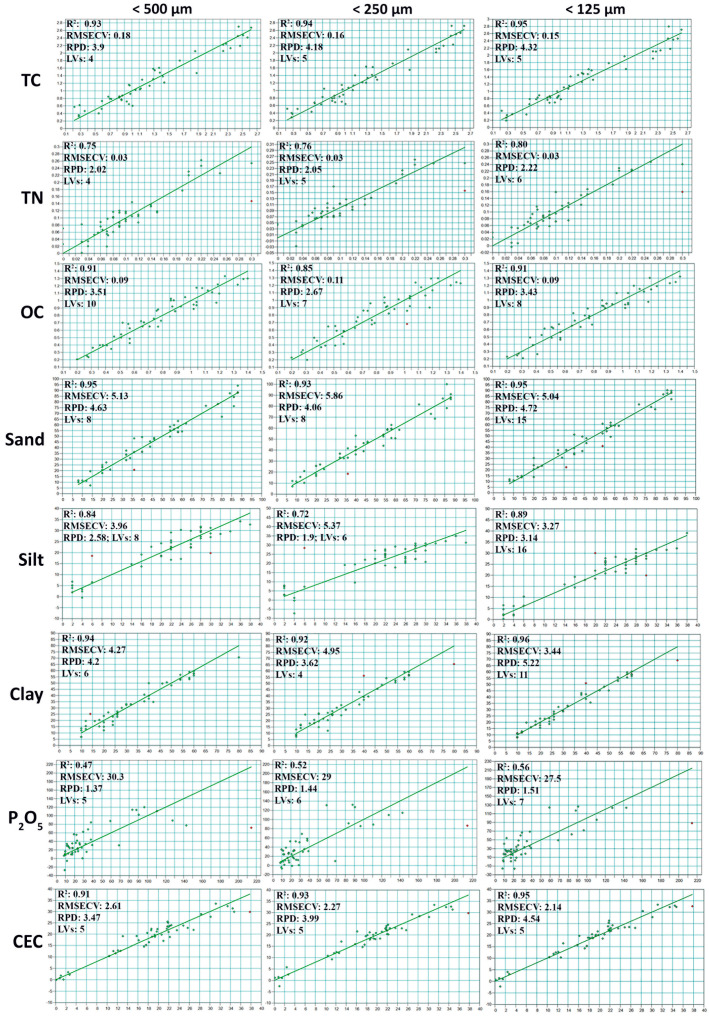
PLSR models of the eight properties of interest, e.g., TC, TN, OC, sand, silt, clay, Olsen P, and CEC, depending on the size of sample particles.

Furthermore, the refinement of the multivariate calibrations, indicated by the improved linearity between the predicted and the real values of the different properties, was directly linked to the increasing correlation coefficients, as shown in Figure 5 and Figure 6.

The total carbon models were upgraded from values of R^2^ = 0.93 and RMSECV = 0.18% with <500 µm samples to values of R^2^ = 0.95 and RMSECV = 0.15% with samples <125 µm; for TN, the predictive capabilities of the PLS models were improved by a value of 0.05 R^2^ units from R^2^ = 0.75 with <500 µm to a value of R^2^ = 0.80; when working with samples lower than 125 µm, the RMSECV value was stable between all the calibrated models at a very low value of 0.03%, and the same trend was noticed for the other properties except for OC, sand, silt, and clay, where the increase in the predictive quality was not significant. The obtained results clarified the improvement in the spectral data quality, translated by the enhancement of the predictive quality of the PLSR models calibrated for the estimation of the different soil properties by making the samples finer after the grinding and sieving operations.

Moreover, the high-quality PLSR model set-up based on the Moroccan soil spectral database (R^2^ > 0.85 and low cross-validation errors) for the seven properties (TC, TN, OC, sand, silt, clay, and CEC) was established, even though the database used in this study contained a limited number of samples (only 50 samples), while, as described in the literature, the larger the databases are, the better the models found will be [33]. This confirms that not only does the size of the database influence the quality of the PLSR models, but also the quality and the representativity of the data components (FTIR spectra) affect the soil spectroscopy models.

**Figure 7:**
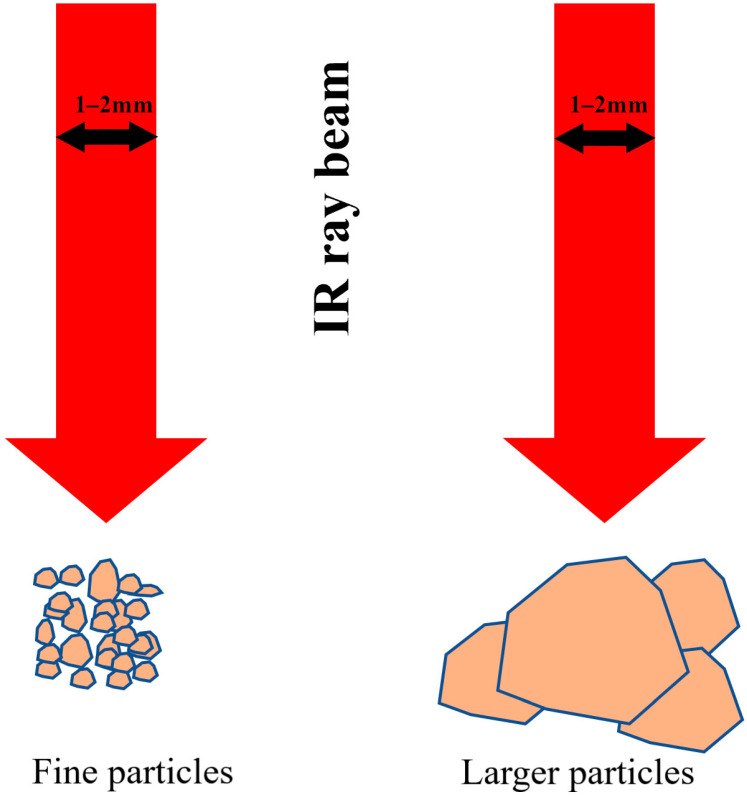
Contact phenomenon between the infrared beam in the FTIR and the soil particles of the sample to be analyzed.

The reason behind this effect can be explained by the contact between the beam diameter of the MIR ray (1–2 mm) and soil particles (Figure 7), the problem with larger particles is that they can cause specular reflections and lead to spectra that correspond to the response of just one particle and do not appropriately represent the sample [12].

## 4. Conclusions

In this paper, it was shown that soil particle size is an essential factor that influences both the quality of FTIR spectra acquisition and affects the multivariate model precision built based on these FTIR spectra for the estimation of eight important soil properties, namely, soil TC, TN, OC, sand, silt, clay, Olsen P, and CEC.

Fifty soil sample have undergone several preparations (drying, grinding, sieving) in order to obtain, at the end, three particle size ranges, i.e., samples with sizes < 500 µm, samples with sizes < 250 µm, and a third range with particles < 125 µm. The PLSR models were set up based on the FTIR spectra recorded on the different obtained samples. A general trend in the improvement of R^2^ as the finer particle was observed (from <500 µm to 125 µm), at least clearly for TC, TN, P_2_O_5_, and CEC, whereas an opposite tendency was observed on the RMSECV. This indicates that the best spectral quality and predictive models were obtained on the finer particles, thus confirming the contact phenomenon theory, such that the finer the particles are, the greater will be the number of particles that will contribute to the acquisition of the FTIR spectrum, and this will lead to a more representative spectrum of the sample.

## Data Availability

The datasets generated and/or analyzed during the current study are not publicly available, but they are available from the corresponding author on reasonable request.
